# Safety and Immunogenicity of the Live-Attenuated Quadrivalent Dengue Vaccine V181 Compared With Butantan-DV Among Healthy Adults in Brazil: A Randomized, Double-Blind, Phase 2 Trial

**DOI:** 10.1093/cid/ciag349

**Published:** 2026-07-01

**Authors:** David Alexanderian, Fabiano Ramos, Soumya Chatterjee, Alexandre Vargas Schwarzbold, Ana Paula Loch, Ariane Cristina Barboza Zanetti, Qiuxu Chen, Jason Martin, Matthew T Onorato, Germán Áñez

**Affiliations:** MRL, Merck & Co., Inc., Rahway, New Jersey, USA; Infectious Diseases Service, Hospital São Lucas da PUC do Rio Grande do Sul, Porto Alegre, Brazil; MRL, Merck & Co., Inc., Rahway, New Jersey, USA; School of Medicine, Department of Health Sciences, Universidade Federal de Santa Maria, Santa Maria, Brazil; Instituto Butantan, São Paulo, Brazil; Instituto Butantan, São Paulo, Brazil; MRL, Merck & Co., Inc., Rahway, New Jersey, USA; MRL, Merck & Co., Inc., Rahway, New Jersey, USA; MRL, Merck & Co., Inc., Rahway, New Jersey, USA; MRL, Merck & Co., Inc., Rahway, New Jersey, USA

**Keywords:** dengue virus, V181, DENV, phase 2, vaccine

## Abstract

**Background:**

Dengue is caused by infection with 1 of 4 dengue virus (DENV) serotypes (DENV-1 to DENV-4). V181 and Butantan-Dengue Vaccine (Butantan-DV) are independently developed, single-dose, quadrivalent dengue vaccines based on the same attenuated parental strains.

**Methods:**

In this randomized, double-blind, active comparator–controlled, multisite, phase 2 trial, we compared the safety and immunogenicity of V181 and Butantan-DV in healthy adults in Brazil. The primary immunogenicity objectives were to compare vaccine-induced neutralizing antibody geometric mean titers (GMTs) and seroconversion rates for DENV-1 to DENV-4 at day 28 postvaccination. The primary and secondary safety objectives, respectively, were to assess vaccine-related serious adverse events and the proportion of participants experiencing solicited adverse events through day 28 postvaccination.

**Results:**

Overall, 1364 participants were assigned (1:1) to receive V181 (n = 682) or Butantan-DV (n = 682). At day 28 postvaccination, V181 induced a noninferior immune response to Butantan-DV for DENV-1 and DENV-2 but not DENV-3 and DENV-4, as measured using GMT ratios. Noninferiority was established for all DENV serotypes based on seroconversion rates. Most participants developed a trivalent or tetravalent neutralizing antibody response. Vaccine viremia was detected for each DENV serotype. V181 and Butantan-DV were generally well tolerated.

**Conclusions:**

V181 was immunogenic in adults, and while immunogenicity was noninferior to Butantan-DV for DENV-1 and DENV-2, seroconversion rates were noninferior for DENV-1 to DENV-4. The safety profile of V181 is comparable to that of Butantan-DV and supports its continued evaluation as a single-dose quadrivalent vaccine for dengue prevention.

**Clinical Trials Registration:**

NCT05710224

## Teaser

V181 was generally well tolerated and immunogenic in adults, demonstrating noninferior immunogenicity to Butantan-DV for dengue virus serotypes 1 and 2 based on neutralizing antibody geometric mean titer ratios and for all 4 dengue virus serotypes, based on seroconversion rates

**(See the Editorial Commentary by Durbin on pages e639–40.)**

Dengue is the most prevalent arthropod-borne viral disease worldwide, leading to substantial morbidity and mortality, and results from infection with any of 4 closely related dengue virus serotypes (*Orthoflavivirus denguei*; DENV-1–4) [1–3]. Each year, ∼390 million infections occur globally, resulting in ∼96 million symptomatic cases and ∼21 000 deaths [4, 5]. Dengue is an increasing concern not only in tropical and subtropical endemic regions, but also in temperate regions reporting autochthonous DENV transmission [6, 7]. Three vaccines are approved for dengue prevention: Dengvaxia®, a 3-dose vaccine [8–10], Qdenga®, a 2-dose vaccine, and recently, Butantan-Dengue Vaccine (Butantan-DV), a single-dose vaccine approved in Brazil [11–13]. There is a substantial need for a single-dose dengue vaccine that provides simultaneous and durable protection against all 4 DENV serotypes, regardless of dengue serostatus [9, 10, 12, 14].

V181 is a quadrivalent dengue vaccine in development for the prevention of disease caused by all 4 DENV serotypes, regardless of prior dengue exposure [14]. V181 comprises attenuated vaccine viral strains that represent all 4 DENV serotypes (rDENV1Δ30, rDENV2/4Δ30(ME), rDENV3Δ30/31, and rDENV4Δ30) [14]. V181 is based on parental strains and quadrivalent TV003 admixture that were originally developed by the US National Institute of Allergy and Infectious Diseases (NIAID) of the National Institutes of Health (NIH) [14, 15]. The parental strains were licensed from the NIAID/NIH and subsequently manufactured and formulated by Merck Sharp & Dohme LLC (a subsidiary of Merck & Co., Inc., Rahway, NJ, USA) [14, 16]. Similarly, Instituto Butantan (São Paulo, Brazil) licensed the same parental strains from the NIAID/NIH to independently manufacture and formulate Butantan-DV [15, 17]. In all 3 vaccine candidates (NIH TV003, V181, and Butantan-DV), each virus component is given at the same dose (∼10^3^ plaque-forming units [PFUs]) [14, 15, 17].

Across phase 1 trials, vaccination with NIH TV003 was generally well tolerated and highly immunogenic [18–20]. Similarly, in a phase 1 trial (V181-001), administration of V181 induced vaccine viremia and robust antibody responses against all 4 DENV serotypes in healthy adults from the United States and Puerto Rico, regardless of prior exposure to orthoflaviviruses, and was generally well tolerated [14]. In a large phase 3 trial (DEN-03-IB) of Brazilian children and adults, Butantan-DV demonstrated efficacy against symptomatic, virologically confirmed dengue due to DENV-1 and DENV-2 through 5 years of follow-up, regardless of baseline dengue serostatus [17, 21, 22].

We compared the immunogenicity and safety of V181 and Butantan-DV in a multisite phase 2 trial of healthy adults in Southern Brazil with historically low dengue rates.

## METHODS

### Study Design

This randomized, double-blind, active comparator-controlled, parallel-group, multisite, phase 2 trial was conducted in healthy adults in Brazil between February 2023 and December 2024 (ClinicalTrials.gov: NCT05710224; protocol V181-002). Participants were assigned (1:1) to receive a subcutaneous single dose (0.5 mL) of V181 or Butantan-DV and were followed for up to 12 months postvaccination (Supplementary Figure 1). Randomization was performed centrally using an Interactive Response Technology system. Both vaccines were supplied open-label and prepared by unblinded site personnel. Participants, investigators, and study personnel remained blinded throughout the study. The study was conducted in accordance with the principles of Good Clinical Practice and approved by the appropriate institutional review boards, biosafety committees, and regulatory agencies. All participants provided written informed consent before enrollment.

To maximize recruitment of dengue-seronegative individuals, a 2-stage strategy was implemented. First, the trial was conducted in regions with historically lower rates of dengue, identified through data from the national dengue surveillance system in Brazil (Sistema de Informação de Agravos de Notificação) and from seroepidemiologic studies conducted in Brazilian populations. Following consent, eligibility based on dengue serostatus was determined using a point-of-care serologic screening assay (Onsite Dengue IgG/IgM Combo Rapid Test; CTK Biotech, CA, USA).

### Study Participants

Healthy adults aged 18–50 years with no known history of dengue or Zika infection were enrolled; full inclusion and exclusion criteria are summarized in Supplementary Table 3.

### Study Vaccines

V181 and Butantan-DV are based on the same attenuated parental strains (rDENV1Δ30, rDENV2/4Δ30[ME], rDENV3Δ30/31, and rDENV4Δ30) and NIH TV003 admixture and were independently manufactured and formulated [14, 15, 17]. A 0.5-mL dose of either vaccine targets delivery of 10^3^ PFUs for each of the vaccine viral components [14, 17].

### Study Objectives

The primary immunogenicity objective was to demonstrate noninferiority of V181 versus Butantan-DV based on vaccine-induced neutralizing antibody (nAb) geometric mean titers (GMTs) and seroconversion rates for each DENV serotype at day 28 postvaccination. The primary safety objective was to evaluate the safety and tolerability of V181 and Butantan-DV based on the proportion of participants experiencing vaccine-related serious adverse events (SAEs) through day 28 postvaccination.

The secondary objective was to evaluate the proportion of participants experiencing solicited adverse events (AEs) through days 5 and 28 postvaccination for injection-site AEs and systemic AEs, respectively.

Exploratory objectives included evaluation of the nAb response rates and GMTs to each DENV serotype at day 28, month 3, and month 12 postvaccination; percentage of vaccine viremia at days 7 and 12 postvaccination; and safety and tolerability of V181 and Butantan-DV based on the proportion of participants experiencing SAEs through month 12 postvaccination.

### Immunogenicity

DENV-nAb titers for each serotype were measured using a validated virus reduction neutralization test (VRNT) [14, 23]. In brief, serially diluted serum samples were mixed separately with each wild-type DENV serotype and incubated with Vero cells. The cells were fixed, stained with serotype-specific DENV primary antibodies, labeled with an Alexa Fluor 448 (Thermo Fisher Scientific, Waltham, MA, USA) secondary antibody, and counted. VRNT_60_ titers were defined as the reciprocal of the highest serum dilution achieving ≥60% neutralization of the wild-type virus compared with the control. The lower limit of quantitation (LLOQ) for each serotype VRNT was as follows: DENV-1, ≥18; DENV-2, ≥15; DENV-3, ≥12; DENV-4, ≥13.

Seroconversion was defined as eliciting a VRNT_60_ titer to each serotype ≥ LLOQ for individuals with titers < LLOQ at baseline. Antibody response rates were defined as the proportion of participants developing a VRNT_60_ titer to each serotype ≥ LLOQ, regardless of their titer at baseline. The DENV-nAb response valency (ie, response to 1, 2, 3, or 4 DENV serotypes [monovalent, bivalent, trivalent, or tetravalent response, respectively]) was determined.

### Vaccine Viremia

Vaccine viremia (ie, RNAemia) was evaluated using a validated quantitative reverse transcription polymerase chain reaction (RT-PCR), as previously described [24]; however, values associated with vaccine viremia were not assessed. In brief, total RNA was extracted from serum samples and run in RT-PCR assays using DENV serotype-specific primer/probes to distinguish between wild-type and vaccine virus. The percentage of participants with serotype-specific vaccine viremia and the valencies of the viremia were determined.

### Safety Assessments

AEs were recorded using an electronic vaccination report card (eVRC) for 28 days postvaccination. Specific injection-site AEs (pain/tenderness, erythema/redness, swelling) were solicited through day 5 postvaccination. Solicited systemic AEs (rash, headache, fatigue, myalgia, arthralgia) were collected through day 28 postvaccination. Additionally, the participants were asked to report daily body temperature, injection-site AEs, and unsolicited AEs via eVRCs through day 28 postvaccination. All medically attended AEs (MAAEs), SAEs, and deaths that occurred from the time of randomization through month 12 postvaccination were reported by the investigator, regardless of whether the events were considered vaccine related. The severity of AEs and laboratory abnormalities was categorized by the investigator as mild (grade 1), moderate (grade 2), severe (grade 3), or potentially life-threatening (grade 4) using prespecified criteria adapted from US Food and Drug Administration (FDA) industry guidance [25]. For solicited injection-site erythema and injection-site swelling, the events were categorized as mild (≤5 cm), moderate (≥5.1 to ≤10 cm), or severe (>10 cm).

All injection-site events were considered vaccine related and, for all systemic AEs, their relation to study intervention was determined by the investigator.

### Statistical Analysis

Immunogenicity assessments were performed on the per-protocol (PP) population, which included all randomized participants without protocol deviations that could have substantially impacted the results of the immunogenicity analyses and excluded participants who were dengue seropositive at baseline, as assessed by VRNT. The safety population was used for safety assessment and included all randomized participants who received study vaccine.

The study was considered to have met its primary immunogenicity objective if V181 was shown to be noninferior to Butantan-DV based on DENV-nAb GMTs and seroconversion rates for each DENV serotype at day 28 postvaccination. The estimation of the nAb GMT ratios to each DENV serotype and the corresponding 95% confidence intervals (CIs) were calculated using a linear model with the log-transformed antibody titer as the response and a single term for vaccination group. Noninferiority as shown by GMTs was demonstrated if the lower bound of the 2-sided 95% CI of the DENV-nAb GMT ratios (V181/Butantan-DV) was >0.67. The between-treatment difference of nAb seroconversion rates to each DENV serotype and its 95% CI were calculated using the Miettinen and Nurminen method [26]. Noninferiority using seroconversion rates was demonstrated if the lower bound of the 2-sided 95% CI for the between-treatment differences (V181 minus Butantan-DV) was >0.1. The primary immunogenicity hypotheses were tested individually for each serotype at a 1-sided 0.025 alpha level. No multiplicity adjustment was required to control the 1-sided type 1 error rate at 0.025. It was estimated that the planned sample size of ∼1240 participants randomized in a 1:1 ratio would provide sufficient power (>95% for GMT, >90% for seroconversion) to assess both primary hypotheses at a 1-sided 0.025 alpha level.

For overall safety endpoints and specific AEs, point estimates and 95% CIs for the differences between treatment groups in the proportion of participants who experienced events were calculated using the Miettinen and Nurminen method [26].

## RESULTS

### Study Population

Overall, 1364 participants were randomized to receive V181 (n = 682) or Butantan-DV (n = 682); 1358 (99.6%) were vaccinated and 1325 (97.1%) completed the study (Figure 1). At day 28, the PP population included 1077 participants (79.0%; V181: 542; Butantan-DV: 535). Reasons for exclusion from the PP population were similar between the study groups (Supplementary Table 4).

**Figure 1.**
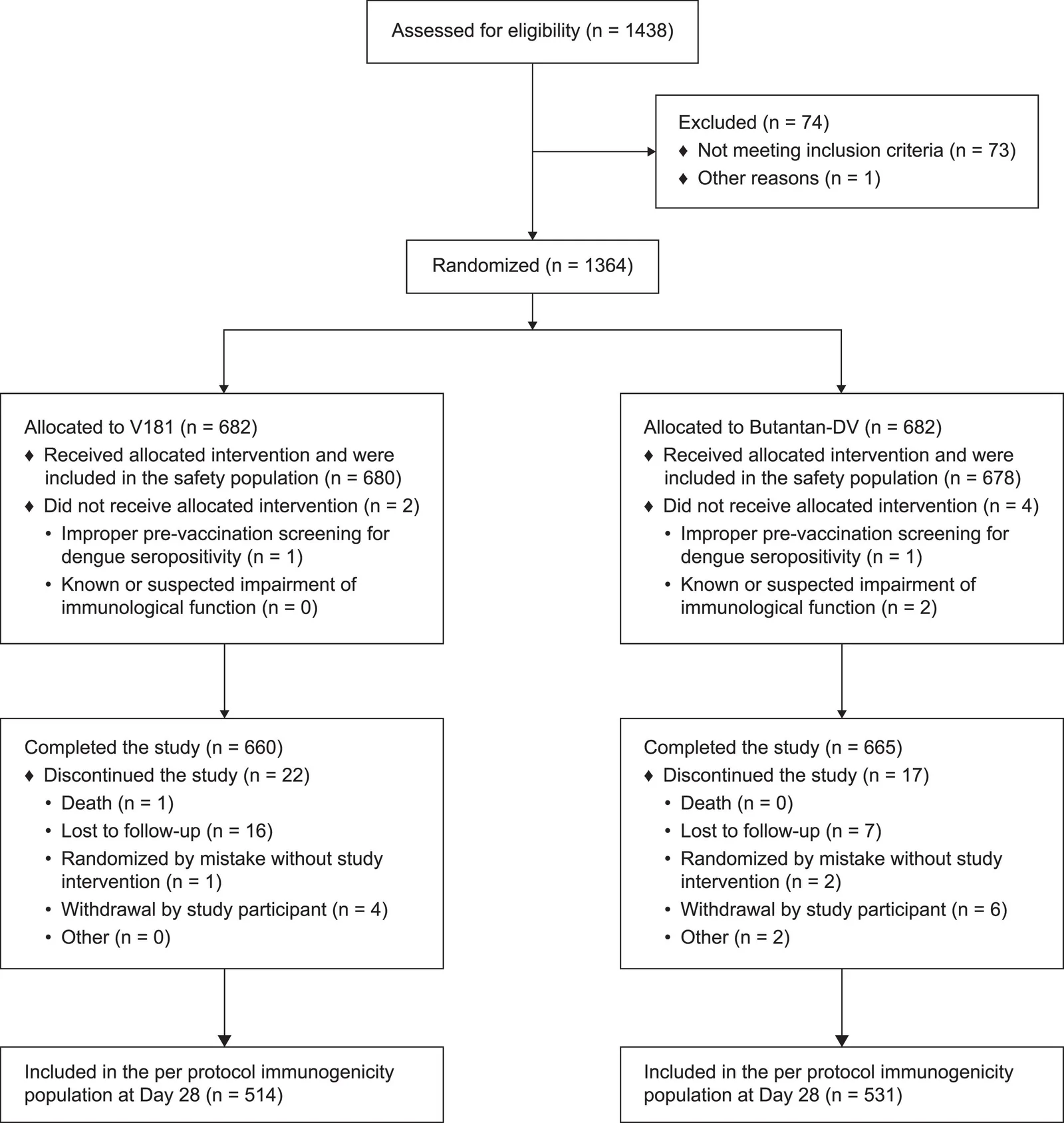
Participant disposition. Abbreviation: DV, dengue vaccine.

There were no major differences in baseline characteristics between the study groups. Most participants were White (85.3%), of Hispanic or Latino ethnicity (83.9%), and female (64.8%). The median age was 31 years, and 93.5% of participants were dengue seronegative at baseline (Table 1). The concordance between the point-of-care IgG/IgM assay and VRNT results was 94.1% (1276/1356).

**Table 1.** Baseline Characteristics (All Randomized Participants)

Characteristic	V181 (n = 682)	Butantan-DV (n = 682)	Total (N = 1364)
Sex, n (%)			
Male	222 (32.6)	257 (37.7)	479 (35.1)
Female	459 (67.3)	425 (62.3)	884 (64.8)
Undifferentiated	1 (0.1)	0	1 (0.1)
Age, y			
18–30, n (%)	316 (46.3)	332 (48.7)	648 (47.5)
31–40, n (%)	197 (28.9)	190 (27.9)	387 (28.4)
41–50, n (%)	169 (24.8)	160 (23.5)	329 (24.1)
Mean (SD)	32.8 (8.9)	32.2 (9.0)	32.5 (9.0)
Median (range)	32.0 (18–50)	31.0 (18–50)	31.0 (18–50)
Race, n (%)			
American Indian or Alaska Native	4 (0.6)	2 (0.3)	6 (0.4)
Asian	3 (0.4)	2 (0.3)	5 (0.4)
Black or African American	70 (10.3)	71 (10.4)	141 (10.3)
Multiracial	20 (2.9)	27 (4.0)	47 (3.4)
White	585 (85.8)	579 (84.9)	1164 (85.3)
Missing	0	1 (0.1)	1 (0.1)
Ethnicity, n (%)			
Hispanic or Latino	569 (83.4)	575 (84.3)	1144 (83.9)
Not Hispanic or Latino	99 (14.5)	92 (13.5)	191 (14.0)
Not reported	4 (0.6)	4 (0.6)	8 (0.6)
Unknown	10 (1.5)	11 (1.6)	21 (1.5)
Serostatus by VRNT, n (%)			
Positive	42 (6.2)	38 (5.6)	80 (5.9)
Negative	638 (93.5)	638 (93.5)	1276 (93.5)
Unknown	2 (0.3)	4 (0.6)	6 (0.4)
Missing	0	2 (0.3)	2 (0.1)

Abbreviations: DV, dengue vaccine; SD, standard deviation; VRNT, virus reduction neutralization test.

### Immunogenicity

#### DENV-nAb GMTs

V181 did not meet the primary objective of demonstrating noninferiority to Butantan-DV for each of the 4 DENV serotypes based on nAb GMTs at day 28 postvaccination. V181 induced a noninferior immune response to Butantan-DV for DENV-1 and DENV-2, but not DENV-3 or DENV-4. The DENV-nAb GMTs for V181 at day 28 postvaccination were comparable to Butantan-DV for DENV-1, higher for DENV-2, and lower for DENV-3 and DENV-4 (Figure 2*A*). GMTs peaked at day 28 and declined gradually through month 12 in both groups (Figure S2A).

**Figure 2.**
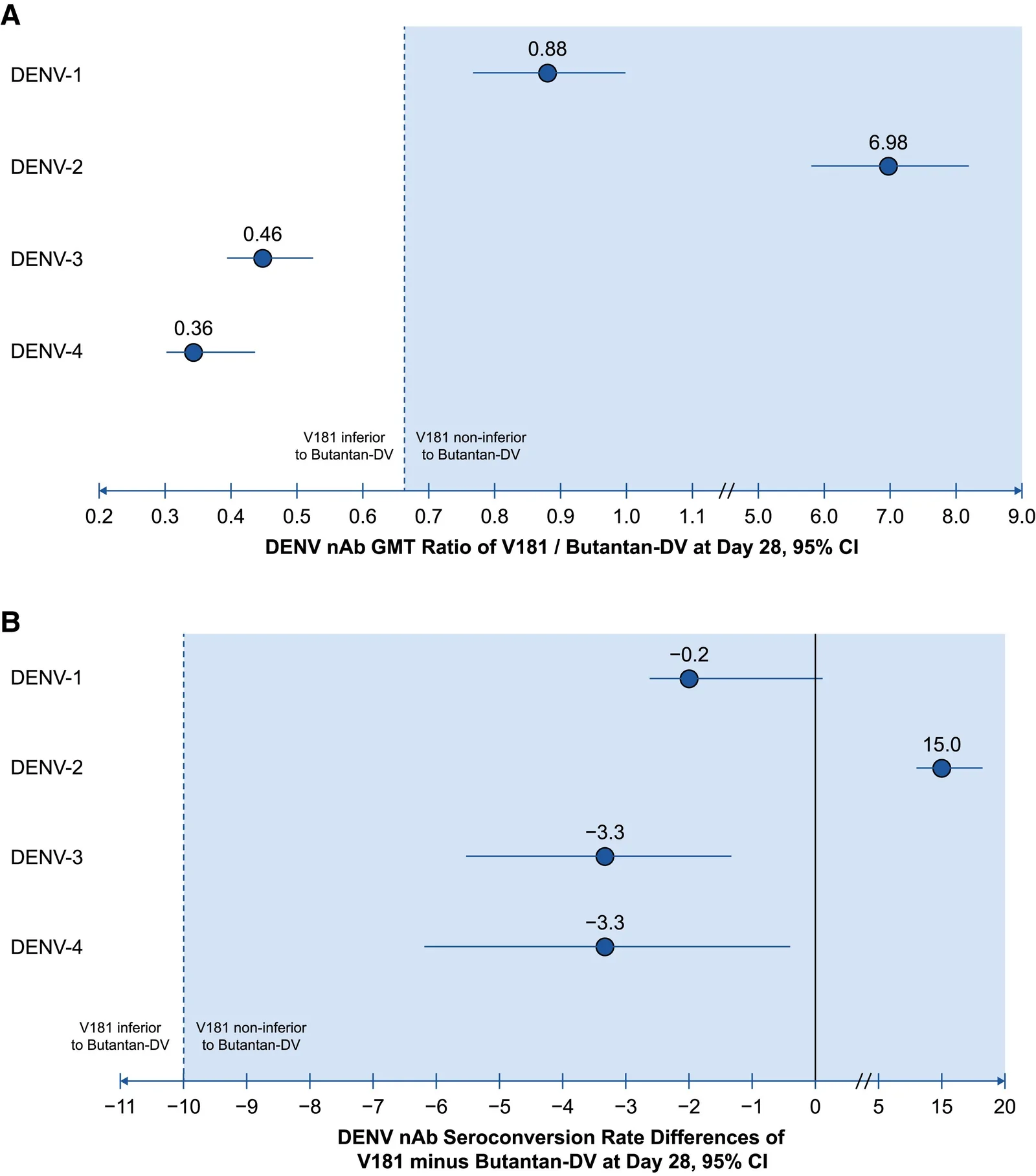
DENV-nAb (*A*) GMT ratios (95% CI; V181/Butantan-DV) and (*B*) seroconversion rates percentage point differences (95% CI; V181 minus Butantan-DV) at day 28 postvaccination (PP population). Abbreviations: CI, confidence interval; DENV, dengue virus; DV, dengue vaccine; GMT, geometric mean titer; nAb, neutralizing antibody; PP, per protocol.

#### DENV-nAb Seroconversion and Response Rates

V181 met the predefined criterion for noninferiority to Butantan-DV for all DENV serotypes, as measured using seroconversion rates at day 28 postvaccination (Figure 2*B*). V181 seroconversion rates at day 28 were comparable to rates with Butantan-DV for DENV-1, DENV-3, and DENV-4 and were higher for DENV-2 (Figure 2*B*). For all DENV serotypes, the observed and cumulative seroconversion rates and nAb response rates were comparable between the V181 and Butantan-DV groups at day 28, month 3, and month 12 postvaccination (Supplementary Figure 2).

#### DENV-nAb Valency

Most participants had a trivalent or tetravalent nAb response at day 28 postvaccination (V181: 96.9%; Butantan-DV: 97.0%; Figure 3). The proportions of participants who had multivalent (response to ≥2 DENV serotypes) responses were comparable between the study groups at any time point (Figure 3).

**Figure 3.**
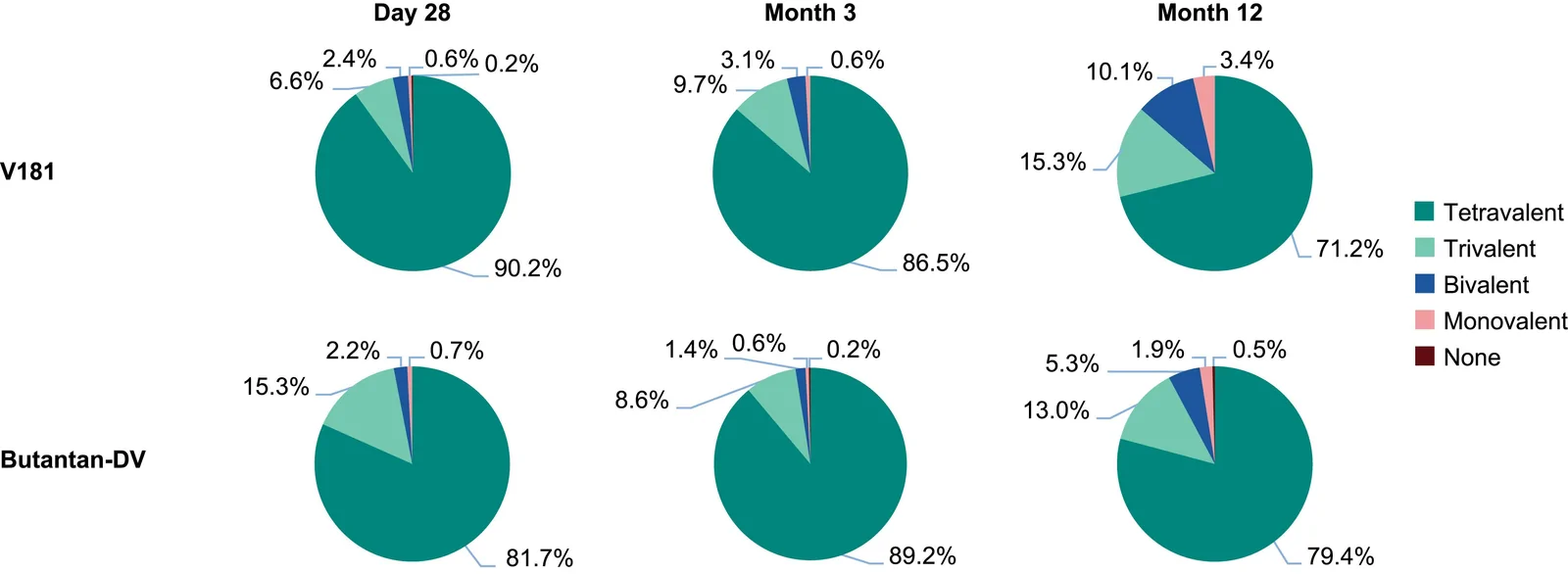
Valency of seropositivity at day 28, month 3, and month 12 postvaccination for V181 and Butantan-DV (PP population). Abbreviations: DV, dengue vaccine; PP, per protocol.

#### Vaccine Viremia

Vaccine viremia for any serotype was detected in 99% of participants in the V181 group and 97% of participants in the Butantan-DV group at any time point (day 7 or day 12; Figure 4*A*). The proportion of participants with vaccine viremia was similar for DENV-1, higher for DENV-2, and lower for DENV-3 and DENV-4 with V181 compared with Butantan-DV (Figure 4*A*). Most participants had multivalent vaccine viremia (V181: 88.4%; Butantan-DV: 85.7%; Figure 4*B*). At any time point, the proportion of participants with monovalent, bivalent, trivalent, or tetravalent DENV viremia was comparable between the groups (Figure 4*B*).

**Figure 4.**
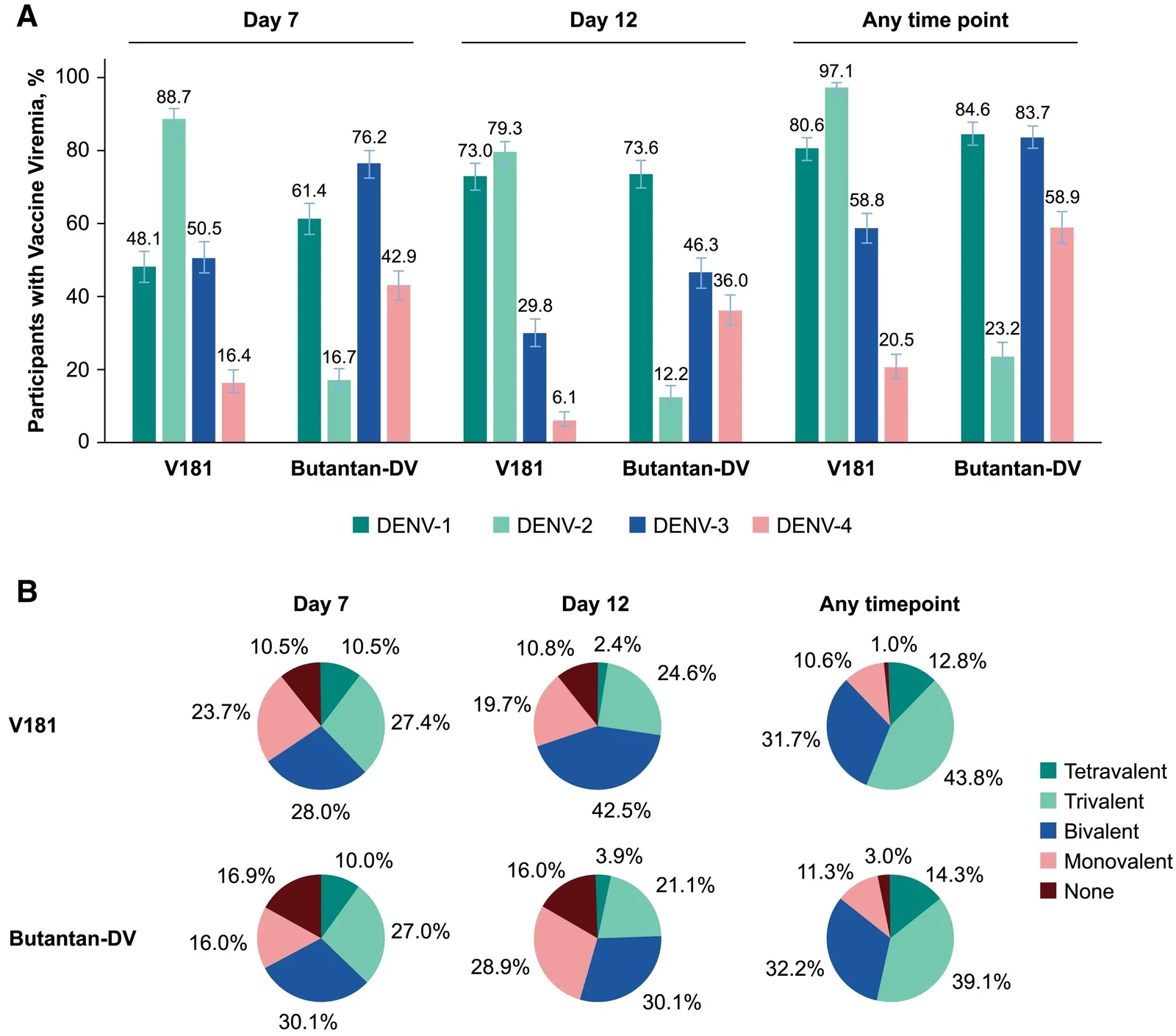
Vaccine viremia (*A*) and valency of vaccine viremia (*B*) at day 7, day 12, and any time point postvaccination for V181 and Butantan-DV (PP population). Abbreviations: DENV, dengue virus; DV, dengue vaccine; PP, per protocol.

### Safety and Tolerability

The proportions of participants who experienced AEs through day 28 postvaccination were comparable between the V181 and the Butantan-DV groups (Table 2); no vaccine-related SAEs occurred in either study group through day 28.

**Table 2.** Adverse Event Summary at Day 28 Postvaccination (Safety Population)

	V181 (n = 680)	Butantan-DV (n = 678)	Total (N = 1358)	Difference In % Versus Butantan-DV Estimate (95% CI)^a^
Participants in population, n (%)				
Who had ≥1 AE	649 (95.4)	637 (94.0)	1286 (94.7)	1.5 (−0.9 to 3.9)
Injection-site	183 (26.9)	168 (24.8)	351 (25.8)	2.1 (−2.5 to 6.8)
Systemic	644 (94.7)	636 (93.8)	1280 (94.3)	0.9 (−1.6 to 3.4)
Who had no AEs	31 (4.6)	41 (6.0)	72 (5.3)	−1.5 (−3.9 to 0.9)
Who had vaccine-related^b^ AEs	615 (90.4)	598 (88.2)	1213 (89.3)	2.2 (−1.1 to 5.6)
Injection-site	183 (26.9)	168 (24.8)	351 (25.8)	2.1 (−2.5 to 6.8)
Systemic	597 (87.8)	585 (86.3)	1182 (87.0)	1.5 (−2.1 to 5.1)
Who had grade 3 AEs	31 (4.6)	47 (6.9)	78 (5.7)	−2.4 (−4.9 to 0.1)
Who had grade 4 AEs	1 (0.1)	1 (0.1)	2 (0.1)	−0.0 (−0.7 to 0.7)
Who had grade 3 vaccine-related AEs	22 (3.2)	40 (5.9)	62 (4.6)	−2.7 (−5.0 to −0.5)
Who had grade 4 vaccine-related AEs	0	0	0	n/a
Who had non-SAEs	649 (95.4)	637 (94.0)	1286 (94.7)	1.5 (−0.9 to 3.9)
Who had SAEs	1 (0.1)	1 (0.1)	2 (0.1)	−0.0 (−0.7 to 0.7)
Who had vaccine-related SAEs	0	0	0	0.0 (−0.6 to 0.6)
Who had medically attended AEs	38 (5.6)	55 (8.1)	93 (6.8)	−2.5 (−5.3 to 0.2)
Who had vaccine-related^b^ medically attended AEs	15 (2.2)	21 (3.1)	36 (2.7)	−0.9 (−2.7 to 0.9)
Who died	0	0	0	0.0 (−0.6 to 0.6)

MedDRA version 27.1 was used in the reporting of this study.

Abbreviations: AE, adverse event; CI, confidence interval; DV, dengue vaccine; MedDRA, Medical Dictionary for Regulatory Activities; n/a, not applicable; SAE, serious adverse event.

^a^Estimated differences and CIs are calculated based on the Miettinen and Nurminen method [26] and are provided in accordance with the statistical analysis plan.

^b^Determined by the investigator to be related to the vaccine.

The proportions of participants who experienced ≥1 solicited injection-site AE through day 5 after V181 or Butantan-DV vaccination were comparable (24.7% and 22.6%, respectively; Table 3). Most participants in the V181 (91.3%) and the Butantan-DV (90.7%) groups experienced ≥1, mostly mild or moderate, solicited systemic AE through day 28 postvaccination (Table 3; Supplementary Table 5). The 3 most frequently reported solicited systemic AEs were rash, headache, and fatigue.

**Table 3.** Solicited Adverse Events Up to Day 28 Postvaccination (Safety Population)

	V181 (n = 680)	Butantan-DV (n = 678)	Total (N = 1358)	Difference In % Versus Butantan-DV Estimate (95% CI)^a^
Participants in population, n (%)				
Who had ≥1 solicited injection-site AE from day 1 through day 5 postvaccination	168 (24.7)	153 (22.6)	321 (23.6)	2.1 (−2.4 to 6.7)
Erythema	70 (10.3)	41 (6.0)	111 (8.2)	4.2 (1.4 to 7.2)
Pain	120 (17.6)	121 (17.8)	241 (17.7)	−0.2 (−4.3 to 3.9)
Swelling	19 (2.8)	15 (2.2)	34 (2.5)	0.6 (−1.1 to 2.3)
Who had ≥1 solicited systemic AE from day 1 through day 28 postvaccination	621 (91.3)	615 (90.7)	1236 (91.0)	0.6 (−2.5 to 3.7)
Arthralgia	139 (20.4)	147 (21.7)	286 (21.1)	−1.2 (−5.6 to 3.1)
Fatigue	293 (43.1)	311 (45.9)	604 (44.5)	−2.8 (−8.1 to 2.5)
Headache	418 (61.5)	438 (64.6)	856 (63.0)	−3.1 (−8.3 to 2.0)
Myalgia	241 (35.4)	294 (43.4)	535 (39.4)	−7.9 (−13.1 to −2.7)
Pyrexia	35 (5.1)	70 (10.3)	105 (7.7)	−5.2 (−8.1 to −2.4)
Rash	480 (70.6)	432 (63.7)	912 (67.2)	6.9 (1.9 to 11.8)

Pyrexia was defined as axillary temperature ≥37.8°C (100.0°F) solicited from day 1 through day 28 postvaccination.

MedDRA version 27.1 was used in the reporting of this study.

Abbreviations: AE, adverse event; CI, confidence interval; DV, dengue vaccine; MedDRA, Medical Dictionary for Regulatory Activities; SAE, serious adverse event.

^a^Estimated differences and CIs are calculated based on the Miettinen and Nurminen method [26] and are provided in accordance with the statistical analysis plan.

Through month 12 postvaccination, the proportions of participants with SAEs or MAAEs were low and generally comparable between groups (Supplementary Table 6). No vaccine-related SAEs were reported, and few participants experienced vaccine-related MAAEs (V181: 2.2%; Butantan-DV: 3.2%). One death of unknown cause (investigated as a possible suicide in a participant with concurrent depression who was receiving treatment at the time of randomization) occurred in the V181 group 155 days after vaccination and was not considered vaccine-related.

## DISCUSSION

In this phase 2 trial, 2 independently manufactured and formulated quadrivalent dengue vaccines (V181 and Butantan-DV) were generally well tolerated and induced broad DENV-specific nAb responses in Brazilian adults, most of whom were DENV seronegative at baseline. V181 did not meet the primary objective of demonstrating a noninferior nAb response versus Butantan-DV against all DENV serotypes based on GMTs. The clinical implications of these results remain uncertain given the lack of a correlate of protection (COP) for dengue vaccines. Notably, V181 elicited a noninferior response for each DENV serotype based on nAb seroconversion rates, with 99.8% of participants (541/542) seroconverting after V181 vaccination.

An ideal dengue vaccine would induce robust nAb responses to all DENV serotypes. V181 and Butantan-DV both elicited broad immunity to all DENV serotypes that was sustained through month 12 postvaccination, consistent with results from a previous phase 2 trial, in which Butantan-DV and NIH TV003 induced robust nAb responses to all DENV serotypes after a single dose [27]. DENV-nAb GMTs for DENV-1 were generally comparable between groups, whereas V181 elicited higher GMTs for DENV-2 and lower GMTs for DENV-3 and DENV-4. This pattern is consistent with findings from a previous phase 1 trial, in which the numerically highest nAb titers after V181 vaccination were directed against DENV-2. Nevertheless, V181, similar to other TV003-based vaccines, elicited robust nAb responses to all DENV serotypes [14]. The clinical significance of the level of vaccine-induced DENV nAb titers remains unknown in the absence of an established COP.

Most participants were dengue seronegative at baseline and seroconverted postvaccination, with a large proportion developing trivalent and tetravalent nAb responses. Consistent with a previous phase 1 study of V181 [14], multivalent vaccine viremia was observed in most participants, reflecting replication of multiple live, attenuated vaccine serotypes in dengue-seronegative individuals.

V181 was generally well tolerated, with a safety profile comparable to that of Butantan-DV and consistent with that previously observed for V181, Butantan-DV, and NIH TV003 [14, 17, 18, 27, 28]. Rash was the most common solicited systemic AE (67.2%) but was predominantly mild and self-limiting, consistent with prior findings [14, 17, 18, 27–29]. The high frequency of rash might be linked to the large proportion of seronegative participants (94.0%), as rash occurs more frequently in DENV-seronegative than DENV-seropositive individuals receiving Butantan-DV [17, 27]. This nonpruritic maculopapular rash is often unnoticed and has been used as a clinical predictor of the ability of NIH admixtures TV003 and TV005 to protect against infection in DENV-2 or DENV-3 human challenge models [29–31]. These findings augment the growing body of clinical evidence supporting the safety profile of these vaccines, particularly among DENV-seronegative individuals.

This study has some limitations. The trial was conducted in a single country, Brazil, and, therefore, may not be generalizable to other populations. The latter part of the study coincided with the early phase of the 2024 dengue outbreak in Rio Grande do Sul [32], where most clinical study sites were located, raising the possibility that unrecognized subclinical infections may have occurred. However, since all day 28 immunogenicity samples were collected by January 2024, it is unlikely that subclinical dengue cases affected the day 28 outcomes. Nevertheless, a potential impact on post–day 28 immunogenicity assessments cannot be excluded and should be considered when interpreting the results. However, the unique enrollment strategy ensured the inclusion of a large proportion of dengue-seronegative participants in a dengue-endemic setting, enabling thorough evaluation of the immunogenicity and safety of V181 in this meaningful population, although the baseline serostatus or vaccination history to orthoflaviviruses other than DENV is unknown. The reasons for the observed differences in the levels of some DENV serotypes nAb titers induced by the 2 independently developed vaccines are unknown but may reflect differences in manufacturing processes and final formulations. Moreover, their clinical relevance cannot be established in the absence of a COP. Finally, although this trial was conducted to evaluate the immunogenicity and safety of V181 and Butantan-DV, vaccine efficacy was not assessed. The efficacy of Butantan-DV was, however, demonstrated against DENV-1 and DENV-2 in a randomized phase 3 trial but could not be assessed against DENV-3 and DENV-4 because of a lack of virologically confirmed dengue cases due to these serotypes [17, 21, 22]. The efficacy of V181 in preventing symptomatic virologically confirmed dengue is being evaluated in an ongoing randomized phase 3 trial (V181-005, MOBILIZE-1) [33].

### Conclusion

V181 and Butantan-DV were immunogenic and generally well tolerated, with comparable safety profiles in adults. V181 met the predefined criterion for noninferiority to Butantan-DV for DENV-1 and DENV-2 but not DENV-3 and DENV-4, as measured by nAb GMT ratios, and for each of the 4 DENV serotypes, as determined by nAb seroconversion rates at day 28 postvaccination. These results highlight the challenges of comparing the immunogenicity across live, attenuated dengue vaccines, even those sharing the same technology and antigenic composition. Nevertheless, these findings support the continued development of V181 as a single-dose dengue vaccine, regardless of prior dengue exposure.

## Supplementary Material

ciag349_Supplementary_Data

## Supplementary Data

Supplementary materials are available at *Clinical Infectious Diseases* online. Consisting of data provided by the authors to benefit the reader, the posted materials are not copyedited and are the sole responsibility of the authors, so questions or comments should be addressed to the corresponding author.
