# Supplementary material for: Safety and Immunogenicity of the Live-Attenuated Quadrivalent Dengue Vaccine V181 Compared With Butantan-DV Among Healthy Adults in Brazil: A Randomized, Double-Blind, Phase 2 Trial
Source: Clin Infect Dis. 2026 Jul 1;83(3):e629–38. doi: 10.1093/cid/ciag349 (PMC13644855; doi:10.1093/cid/ciag349)
Supplement: Supplementary file 1 [file ciag349_supplementary_data.docx]

**SUPPLEMENTARY MATERIALS**

**Figure S1.** Study design. ^a^Participants who report rash onset from day 1 through day 28 postvaccination should contact the site promptly to ensure that the rash is assessed by the investigator within 72 hours of onset. If rash assessment cannot occur at a planned visit (visit 2, 3, or 4) within 72 hours of rash onset, the rash should be assessed by the investigator at an unscheduled visit.

^b^Participants who experience protocol-specified fever (axillary temperature of
≥37.8 °C) for ≥2 consecutive days postvaccination at any time during the study should schedule an initial evaluation for suspected Dengue as soon as possible and no later than 8 days after the second day of protocol-specified fever. Abbreviations: DV, dengue vaccine; eVRC, electronic vaccination report card; MAAE, medically attended adverse event; N, number of participants; n, number of participants in the vaccination group; SAE, serious adverse event.

**Figure S2.** DENV-neutralizing antibody GMTs (A), seroconversion rates (B), and antibody response rates (C) at day 28, month 3, and month 12 postvaccination (PP population). DENV, dengue virus; GMT, geometric mean titer; PP, per protocol.

**A**

**B**

**C**

**Table S1.** V181-002 Study Team

| **Name** | **Institution** |
| --- | --- |
| Jeffrey Nickel | Merck & Co., Inc., Rahway, NJ, USA |
| Lourdes Rodriguez | Merck & Co., Inc., Rahway, NJ, USA |
| Noorussabah Shaikh-Shahid | Merck & Co., Inc., Rahway, NJ, USA |
| Scott Gilbert | Merck & Co., Inc., Rahway, NJ, USA |
| Jeffrey Lavenberg | Merck & Co., Inc., Rahway, NJ, USA |
| Sabrina Gozlan Kelner | Merck & Co., Inc., Rahway, NJ, USA |
| Beth-Ann G. Coller | Merck & Co., Inc., Rahway, NJ, USA |
| Luis Castagnini | Merck & Co., Inc., Rahway, NJ, USA |
| Androniki Bili | Merck & Co., Inc., Rahway, NJ, USA |
| Lucy Li | Merck & Co., Inc., Rahway, NJ, USA |
| Fernanda Castro Boulos | Fundação Butantan, São Paulo, Brazil |
| José Alfredo De Sousa Moreira | Fundação Butantan, São Paulo, Brazil |
| Sâmia Silveira Souza Teixeira | Fundação Butantan, São Paulo, Brazil |
| Vanessa Infante | Fundação Butantan, São Paulo, Brazil |
| Maria Beatriz Bastos Lucchesi | Fundação Butantan, São Paulo, Brazil |
| Teresa Sukiennik | Irmandade da Santa Casa de Misericórdia de Porto Alegre, Porto Alegre, Rio Grande do Sul, Brazil |
| Mauro Waldemar Keiserman | LMK Serviços Médicos S/S-Reumacenter, Porto Alegre, Rio Grande do Sul, Brazil |
| Fabio Andre Franke | ONCOSITE – Centro de Pesquisa  Clinica em Oncologia, Ijuí Rio Grande do Sul, Brazil |
| Isabele Berti | Hospital Tacchini, Bento Goncalves, Rio Grande do Sul, Brazil |
| Alexandre Pereira Tognon | Hospital São Vicente de Paulo-Education and Research Management,  Passo Fundo, Rio Grande do Sul, Brazil |
| Felipe Dal Pizzol | Hospital São José de Criciúma, Criciuma, Santa Catarina, Brazil |
| Danise Senna Oliveira | Hospital Escola da Universidade, Federal de Pelotas, Pelotas, Rio Grande do Sul, Brazil |
| Marcela Santiago Biernat | Núcleo de Pesquisa Clínica do Rio Grande do Sul, Porto Alegre, Rio Grande do Sul, Brazil |
| Rafaela Kuczynski Da Rocha | Hospital São Lucas da PUC do Rio Grande do Sul, Porto Alegre, Brazil |
| Fernanda Marcolla Weber | Fundação Universidade de Caxias do Sul (FUCS)-Instituto de Pesquisas em Saúde (IPS), Caxias do Sul, Rio Grande do Sul, Brazil |
| Cristiano Devenci Vendrame | Clínica Supera, Chapeco, Santa Catarina, Brazil |
| Julio Cesar Stobbe | Instituto Méderi de Pesquisa e Saúde, Passo Fundo, Rio Grande do Sul, Brazil |
| Paulo Ernesto Gewehr Filho | Hospital Moinhos de Vento-Centro de Pesquisa Clínica, Porto Alegre, Rio Grande do Sul, Brazil |

**Table S2.** List of Investigators

| **Principal Investigator^a^** | **Institution** |
| --- | --- |
| Alexandre Vargas Schwarzbold | Universidade Federal de Santa Maria -Centro de Pesquisa Clínica-CPC, Av. Roraima 1000  Santa Maria, Rio Grande do Sul, 97105-900, Brazil |
| Teresa Sukiennik | Irmandade da Santa Casa de Misericórdia de Porto Alegre-Centro de Pesquisa em Infectologia, 155, Avenida Independência Centro Histórico, Porto Alegre, Rio Grande do Sul, 90035-074, Brazil |
| Mauro Waldemar Keiserman | LMK Serviços Médicos S/S-Reumacenter, Av. Carlos Gomes 328, Cj.1008, Porto Alegre, Rio Grande do Sul, 90480-000, Brazil |
| Fabio Andre Franke | ONCOSITE – Centro de Pesquisa  Clinica em Oncologia, RUA SAO CRISTOVAO 618 Ijui, Rio Grande do Sul 98700-000, Brazil |
| Isabele Berti | Hospital Tacchini, Rua General Osorio 235, Bento Goncalves, Rio Grande do Sul 95700-000, Brazil |
| Alexandre Pereira Tognon | Hospital São Vicente de Paulo-Education and Research Management, Rua Teixeira Soares, 808, 4º Floor - Gerência de Ensino e Pesquisa  Passo Fundo, Rio Grande do Sul, 99010080, Brazil |
| Felipe Dal Pizzol | Hospital São José de Criciúma, Cel Pedro Benedet, 630 Criciúma, Santa Catarina 88811-  508, Brazil |
| Danise Senna Oliveira | Hospital Escola da Universidade, Federal de Pelotas, Professor Araujo, 538 Pelotas, Rio Grande do Sul, 96015-000, Brazil |
| Marcela Santiago Biernat | Núcleo de Pesquisa Clínica do Rio Grande do Sul, QUINTINO BOCAIUVA 15051002, Porto Alegre, Rio Grande do Sul, Brazil |
| Fabiano Ramos/Rafaela Kuczynski Da Rocha | Hospital São Lucas da PUCRS-Centro  de Pesquisa Clínica HSL-PUCRS, Avenida Ipiranga, 6690, 4 Andar, Porto Alegre, Rio Grande do Sul, 90610-000, Brazil |
| Fernanda Marcolla Weber | Fundação Universidade de Caxias do Sul (FUCS)-Instituto de Pesquisas em Saúde (IPS)  Rua Francisco Getulio Vargas, 1130, Bloco S, Sala 315, Caxias do Sul, Rio Grande do Sul, 95070560, Brazil |
| Cristiano Devenci Vendrame | Clínica Supera, 1259 Rua São Marcos, Chapeco, Santa Catarina 89812-211, Brazil |
| Julio Cesar Stobbe | Instituto Méderi de Pesquisa e Saúde, Av. Sete de Setembro, 55, Passo Fundo, Rio Grande do Sul, 99010-120, Brazil |
| Paulo Ernesto Gewehr Filho | Hospital Moinhos de Vento-Centro de Pesquisa Clínica, 910 Rua Ramiro Barcelos, Porto Alegre, Rio Grande do Sul, 90035-001, Brazil |

^a^If the original investigator has been replaced, that investigator's name is followed by a slash (/) and the replacement investigator's name.

**Table S3.**  Participant eligibility criteria

| **Inclusion criteria** | Per the study protocol, a participant was eligible for inclusion in the study if the participant: |
| --- | --- |
| *Type of participant and disease characteristics* | Was healthy based on medical history and physical examination, according to the clinical judgment of the investigator |
| *Demographics* | Was male or female, from 18 years to 50 years of age, inclusive, at the time of signing the informed consent |
| *Male participants* | Male participants were eligible to participate if they agreed to the following for at least 90 days after administration of study intervention:   - Be abstinent from heterosexual intercourse as their preferred and usual lifestyle (abstinent on a long-term and persistent basis) and agree to remain abstinent   OR   - Must agree to use contraception unless confirmed to be azoospermic - Contraceptive use by men should be consistent with local regulations regarding the methods of contraception for those participating in clinical studies |
| *Female participants* | Female participants were eligible to participate if they were not pregnant or breastfeeding, and at least one of the following conditions applied:   - Were not a woman of childbearing potential (WOCBP)   OR   - Were a WOCBP and using a contraceptive method that is highly effective, or remained abstinent from heterosexual intercourse as their preferred and usual lifestyle (abstinent on a long-term and persistent basis) for at least 90 days after administration of study intervention - A WOCBP had to have had a negative highly sensitive pregnancy test (urine or serum, as required by local regulations) before administration of study intervention - If a urine test could not be confirmed as negative (eg, an ambiguous result), a serum pregnancy test was required. In such cases, the participant was excluded from participation if the serum pregnancy result was positive - The investigator was responsible for review of medical history, menstrual history, and recent sexual activity to decrease the risk for inclusion of a woman with an early undetected pregnancy - Contraceptive use by women should be consistent with local regulations regarding the methods of contraception for those participating in clinical studies |
| *Informed consent* | The participant (or legally acceptable representative) had provided documented informed consent for the study |
| *Additional categories* | Was dengue seronegative based on a prevaccination point-of-case dengue test |
| **Exclusion criteria** | Per the study protocol, a participant was excluded from the study if the participant: |
| *Medical conditions* | Had a known history of dengue or Zika natural infection |
|  | Had an acute febrile illness (axillary temperature ≥37.8°C) occurring within 72 hours prior to receipt of study vaccine |
|  | Had a known hypersensitivity or history of severe allergic reaction (eg, swelling of the mouth and throat, difficulty breathing, hypotension, or shock) to any component of the dengue vaccine, that required medical intervention |
|  | Had a serious or progressive disease according to the investigator, including but not limited to cancer, uncontrolled diabetes, severe cardiac, renal or hepatic insufficiency, systemic autoimmune disease, or neurologic disorder |
|  | Had known or suspected impairment of immunological function, including, but not limited to congenital or acquired immunodeficiency, HIV infection, hematologic malignancy, or treatment for autoimmune diseases |
|  | Had a condition in which repeated venipuncture or injections pose more than minimal risk for the participant, such as hemophilia, thrombocytopenia, other severe coagulation disorders, or significantly impaired venous access |
| *Prior/concomitant therapy* | Had received a dose of any dengue vaccine (investigational or approved) prior to study entry, or planned to receive any dengue vaccine (investigational or approved) for the duration of the trial |
|  | Had received a licensed non-live vaccine within 14 days before receipt of study vaccine or was scheduled to receive any licensed non-live vaccine within 28 days following receipt of study vaccine. Exception: Inactivated influenza vaccine could be administered, but must have been given at least 7 days before receipt of study vaccine or at least 28 days after receipt of study vaccine |
|  | Had received a licensed live vaccine within 28 days prior to receipt of study vaccine or was scheduled to receive any live vaccine within 28 days following receipt of study vaccine |
|  | Had received systemic corticosteroids (equivalent of ≥2 mg/kg/day of prednisone or ≥20 mg/day for persons weighing >10 kg) for ≥14 consecutive days and had not completed treatment at least 30 days before study entry or was expected to receive systemic corticosteroids at aforementioned dose and duration within 28 days following receipt of study vaccine. (Note: topical and inhaled/nebulized steroids were permitted) |
|  | Had received systemic corticosteroids exceeding physiologic replacement doses (approximately 5 mg/day prednisone equivalent) within 14 days before vaccination |
|  | Had received immunosuppressive therapies, including chemotherapeutic agents used to treat cancer or other conditions, treatments associated with organ or bone marrow transplantation, or autoimmune disease, within 6 months prior to receipt of study vaccine, or planned to receive immunosuppressive therapies within 28 days following receipt of study vaccine |
|  | Had received a blood transfusion or blood products (including immunoglobulins) within 6 months prior to receipt of study vaccine or planned to receive a blood transfusion or blood products (including immunoglobulins) within 28 days following receipt of study vaccine |
| *Prior/concurrent clinical study experience* | Had participated in another interventional clinical study within 6 months prior to signing the informed consent or planned to participate in another interventional clinical study at any time during the duration of the current clinical study. Participants enrolled in observational studies were eligible to be included; these were reviewed on a case-by-case basis for approval by the Sponsor |
| *Other exclusions* | Had any other reason that, in the opinion of the investigator, may interfere with the evaluations required by the study. Reasons included, but were not limited to, being unable to complete the eVRC, being unable to complete visits including non-office visit contacts (eg, telephone), or being unable to comply with study procedures |
|  | Had planned donation of blood, eggs, or sperm at any time from signing the informed consent through 90 days postvaccination |
|  | Was or had an immediate family member (eg, spouse, parent/legal guardian, sibling, or child) who is investigational site or Sponsor staff directly involved with this study |

Abbreviations: eVCR, electronic Vaccination Report Card.

**Table S4.** Participant Inclusion for Immunogenicity Analysis (PP Population)

|  | **V181**  **(n = 682)** | **Butantan-DV**  **(n = 682)** | **Total**  **(N = 1364)** |
| --- | --- | --- | --- |
| Participants included in analyses by time point, n (%) |  |  |  |
| Day 1 | 576 (84.5) | 566 (83.0) | 1142 (83.7) |
| Day 28 | 542 (79.5) | 535 (78.4) | 1077 (79.0) |
| Month 3 | 513 (75.2) | 510 (74.8) | 1023 (75.0) |
| Month 12 | 386 (56.6) | 378 (55.4) | 764 (56.0) |
| Reasons for exclusions from analysis^a^, n (%) |  |  |  |
| Participant-level exclusions^b^ | 103 (15.1) | 116 (17.0) | 219 (16.1) |
| Randomly assigned but not vaccinated | 2 (0.3) | 4 (0.6) | 6 (0.4) |
| Study intervention improperly stored | 5 (0.7) | 18 (2.6) | 23 (1.7) |
| Seropositive for any of the 4 dengue serotypes by VRNT at baseline | 42 (6.2) | 38 (5.6) | 80 (5.9) |
| Baseline serostatus unknown by VRNT | 2 (0.3) | 4 (0.6) | 6 (0.4) |
| Impairment of immune function | 0 | 2 (0.3) | 2 (0.1) |
| Received prohibited vaccine | 1 (0.1) | 0 | 1 (0.1) |
| Received prohibited systemic corticosteroid | 1 (0.1) | 0 | 1 (0.1) |
| Pregnant or breastfeeding | 1 (0.1) | 1 (0.1) | 2 (0.1) |
| Missing serology results | 0 (0.0) | 2 (0.3) | 2 (0.1) |
| Immunogenicity sample clotting errors at visit 1 | 62 (9.1) | 55 (8.1) | 117 (8.6) |
| Visit-level exclusions—day 1 | 3 (0.4) | 0 | 3 (0.2) |
| Hemolyzed lipemic or icteric sample | 3 (0.4) | 0 | 3 (0.2) |
| Visit-level exclusions—day 28 | 37 (5.4) | 31 (4.5) | 68 (5.0) |
| Hemolyzed lipemic or icteric sample | 1 (0.1) | 0 | 1 (0.1) |
| Immunogenicity blood draw outside window | 18 (2.6) | 18 (2.6) | 36 (2.6) |
| Missing serology results | 2 (0.3) | 4 (0.6) | 6 (0.4) |
| Immunogenicity sample clotting errors at visit 4 | 8 (1.2) | 3 (0.4) | 11 (0.8) |
| Any dengue cases^c^ | 4 (0.6) | 2 (0.3) | 6 (0.4) |
| Received prohibited vaccine | 6 (0.9) | 5 (0.7) | 11 (0.8) |
| Visit-level exclusions—month 3 | 66 (9.7) | 56 (8.2) | 122 (8.9) |
| Hemolyzed lipemic or icteric sample | 29 (4.3) | 24 (3.5) | 53 (3.9) |
| Immunogenicity blood draw outside window | 27 (4.0) | 20 (2.9) | 47 (3.4) |
| Missing serology results | 5 (0.7) | 4 (0.6) | 9 (0.7) |
| Immunogenicity sample clotting errors at visit 5 | 1 (0.1) | 6 (0.9) | 7 (0.5) |
| Any dengue cases^c^ | 4 (0.6) | 2 (0.3) | 6 (0.4) |
| Visit-level exclusions—month 12 | 193 (28.3) | 188 (27.6) | 381 (27.9) |
| Hemolyzed lipemic or icteric sample | 101 (14.8) | 87 (12.8) | 188 (13.8) |
| Immunogenicity blood draw outside window | 80 (11.7) | 89 (13.0) | 169 (12.4) |
| Missing serology results | 17 (2.5) | 14 (2.1) | 31 (2.3) |
| Immunogenicity sample clotting errors at visit 8 | 2 (0.3) | 3 (0.4) | 5 (0.4) |
| Any dengue cases^c^ | 5 (0.7) | 2 (0.3) | 7 (0.5) |

Percentages were calculated based on the number of randomly assigned participants.

Abbreviations: ELISA, enzyme-linked immunosorbent assay; NS, nonstructural protein; PP, per protocol; RT-PCR, reverse transcription polymerase chain reaction; VRNT, virus reduction neutralization test.

^a^Participants may have >1 reason for exclusion.

^b^Participant-level exclusions result in exclusion from analysis at all time points. These participants do not also appear in the visit-level exclusions rows.

^c^Defined as participant with positive, indeterminate, not collected, or not tested sample results for quantitative dengue serotype-specific RT-PCR assay or NS1 ELISA.

**Table S5.** Solicited Adverse Events by Grade (Safety Population)

|  | **V181**  **(n = 680)** | **Butantan-DV**  **(n = 678)** | **Total**  **(N = 1358)** |
| --- | --- | --- | --- |
| Participants in population, n (%) |  |  |  |
| With ≥1 solicited systemic AEs, n (%) | 621 (91.3) | 615 (90.7) | 1236 (91.0) |
| Mild (grade 1) | 344 (50.6) | 292 (43.1) | 636 (46.8) |
| Moderate (grade 2) | 254 (37.4) | 279 (41.2) | 533 (39.2) |
| Severe (grade 3) | 23 (3.4) | 41 (6.0) | 64 (4.7) |
| Potentially life threatening (grade 4) | 0 | 0 | 0 |
| Not assigned | 0 | 3 (0.4) | 3 (0.2) |
| Arthralgia^a^ | 139 (20.4) | 147 (21.7) | 286 (21.1) |
| Mild (grade 1) | 85 (12.5) | 87 (12.8) | 172 (12.7) |
| Moderate (grade 2) | 50 (7.4) | 54 (8.0) | 104 (7.7) |
| Severe (grade 3) | 4 (0.6) | 6 (0.9) | 10 (0.7) |
| Fatigue^a^ | 293 (43.1) | 311 (45.9) | 604 (44.5) |
| Mild (grade 1) | 198 (29.1) | 198 (29.2) | 396 (29.2) |
| Moderate (grade 2) | 90 (13.2) | 104 (15.3) | 194 (14.3) |
| Severe (grade 3) | 5 (0.7) | 9 (1.3) | 14 (1.0) |
| Headache^a^ | 418 (61.5) | 438 (64.6) | 856 (63.0) |
| Mild (grade 1) | 252 (37.1) | 223 (32.9) | 475 (35.0) |
| Moderate (grade 2) | 157 (23.1) | 200 (29.5) | 357 (26.3) |
| Severe (grade 3) | 9 (1.3) | 15 (2.2) | 24 (1.8) |
| Myalgia^a^ | 241 (35.4) | 294 (43.4) | 535 (39.4) |
| Mild (grade 1) | 158 (23.2) | 180 (26.5) | 338 (24.9) |
| Moderate (grade 2) | 78 (11.5) | 107 (15.8) | 185 (13.6) |
| Severe (grade 3) | 5 (0.7) | 7 (1.0) | 12 (0.9) |
| Pyrexia^a,b^ | 35 (5.1) | 70 (10.3) | 105 (7.7) |
| Mild (grade 1) | 10 (1.5) | 22 (3.2) | 32 (2.4) |
| Moderate (grade 2) | 8 (1.2) | 18 (2.7) | 26 (1.9) |
| Severe (grade 3) | 6 (0.9) | 14 (2.1) | 20 (1.5) |
| Not assigned | 11 (1.6) | 16 (2.4) | 27 (2.0) |
| Rash^a^ | 480 (70.6) | 432 (63.7) | 912 (67.2) |
| Mild (grade 1) | 392 (57.6) | 336 (49.6) | 728 (53.6) |
| Moderate (grade 2) | 86 (12.6) | 92 (13.6) | 178 (13.1) |
| Severe (grade 3) | 2 (0.3) | 4 (0.6) | 6 (0.4) |

Abbreviations: AE, adverse event; DV, dengue vaccine; FDA, US Food and Drug Administration; MedDRA, Medical Dictionary for Regulatory Activities.

Every participant was counted once for each applicable specific AE and is classified according to the highest nonmissing grading.

Grades are adapted from and aligned with the FDA Guidance for Industry, September 2007 [24].

MedDRA version 27.1 was used in the reporting of this study.

^a^Arthralgia, fatigue, myalgia, headache, pyrexia, and rash were solicited from day 1 through day 28 postvaccination.

^b^Pyrexia was defined as axillary temperature ≥37.8 °C (100.0 °F) solicited from day 1 through day 28 postvaccination. Pyrexia from day 1 through day 28 postvaccination is graded according to maximum temperature and presented as follows: 38.0 °C (100.4 °F) to <38.5 °C (101.2 °F) = mild; 38.5 °C (101.2 °F) to <39.0 °C (102.1 °F) = moderate; 39.0 °C (102.1 °F) to ≤40.0 °C (104.0 °F) = severe; >40.0 °C (104.0 °F) = potentially life threatening. The intensity grade for pyrexia with elevated temperatures was determined based on participant-reported temperature in original unit. Participants with a temperature of 37.8 °C (100.0 °F) to <38.0 °C (100.4 °F) were not assigned a grade for pyrexia.

**Table S6.** Safety and Tolerability Through Month 12 Postvaccination (Safety Population)

|  | **V181**  **(n = 680)** | **Butantan-DV**  **(n = 678)** | **Total**  **(N = 1358)** | **Difference in % V181 vs Butantan-DV**  **Estimate (95% CI)^a^** |
| --- | --- | --- | --- | --- |
| Participants in population, n (%) | | | | |
| Who had ≥1 AEs | 650 (95.6) | 636 (93.8) | 1286 (94.7) | 1.8 (−0.6 to 4.2) |
| Injection-site | 183 (26.9) | 168 (24.8) | 351 (25.8) | 2.1 (−2.5 to 6.8) |
| Systemic | 645 (94.9) | 635 (93.7) | 1280 (94.3) | 1.2 (−1.3 to 3.7) |
| Who had no AEs | 30 (4.4) | 42 (6.2) | 72 (5.3) | −1.8 (−4.2 to 0.6) |
| Who had vaccine-related^b^ AEs | 617 (90.7) | 598 (88.2) | 1215 (89.5) | 2.5 (−0.7 to 5.8) |
| Injection-site | 183 (26.9) | 168 (24.8) | 351 (25.8) | 2.1 (−2.5 to 6.8) |
| Systemic | 599 (88.1) | 585 (86.3) | 1184 (87.2) | 1.8 (−1.8 to 5.4) |
| Who had grade 3 AEs | 33 (4.9) | 50 (7.4) | 83 (6.1) | −2.5 (−5.2 to 0.03) |
| Who had grade 4 AEs | 13 (1.9) | 9 (1.3) | 22 (16.2) | 0.6 (−0.8 to 2.1) |
| Who had grade 3 vaccine-related AEs | 22 (3.2) | 41 (6.0) | 63 (4.6) | −2.8 (−5.2 to −0.6) |
| Who had grade 4 vaccine-related AEs | 0 | 0 | 0 | n/a |
| Who had non-SAEs | 650 (95.6) | 636 (93.8) | 1,286 (94.7) | 1.8 (−0.6 to 4.2) |
| Who had SAEs | 12 (1.8) | 9 (1.3) | 21 (1.5) | 0.4 (−1.0 to 1.9) |
| Who had vaccine-related SAEs | 0 | 0 | 0 | 0.0 (−0.6 to 0.6) |
| Who had medically attended AEs | 125 (18.4) | 149 (22.0) | 274 (20.2) | −3.6 (−7.9 to 0.7) |
| Who had vaccine-related^b^ medically  attended AEs | 15 (2.2) | 22 (3.2) | 37 (2.7) | −1.0 (−2.9 to 0.7) |
| Who died | 1 (0.1) | 0 | 1 (0.1) | 0.1 (−0.4 to 0.8) |

Abbreviations: AE, adverse event; CI, confidence interval; DV, dengue vaccine; n/a, not applicable; SAE, serious adverse event.

^a^Estimated differences and CIs are calculated based on the Miettinen and Nurminen method [25] and are provided in accordance with the statistical analysis plan.

^b^Determined by the investigator to be related to the vaccine.
